# Co-Expression of Two Subtypes of Melatonin Receptor on Rat M1-Type Intrinsically Photosensitive Retinal Ganglion Cells

**DOI:** 10.1371/journal.pone.0117967

**Published:** 2015-02-25

**Authors:** Wen-Long Sheng, Wei-Yi Chen, Xiong-Li Yang, Yong-Mei Zhong, Shi-Jun Weng

**Affiliations:** Institute of Neurobiology, Institutes of Brain Science, State Key Laboratory of Medical Neurobiology and Collaborative Innovation Center for Brain Science, Fudan University, Shanghai, China; Dalhousie University, CANADA

## Abstract

Intrinsically photosensitive retinal ganglion cells (ipRGCs) are involved in circadian and other non-image forming visual responses. An open question is whether the activity of these neurons may also be under the regulation mediated by the neurohormone melatonin. In the present work, by double-staining immunohistochemical technique, we studied the expression of MT_1_ and MT_2_, two known subtypes of mammalian melatonin receptors, in rat ipRGCs. A single subset of retinal ganglion cells labeled by the specific antibody against melanopsin exhibited the morphology typical of M1-type ipRGCs. Immunoreactivity for both MT_1_ and MT_2_ receptors was clearly seen in the cytoplasm of all labeled ipRGCs, indicating that these two receptors were co-expressed in each of these neurons. Furthermore, labeling for both the receptors were found in neonatal M1 cells as early as the day of birth. It is therefore highly plausible that retinal melatonin may directly modulate the activity of ipRGCs, thus regulating non-image forming visual functions.

## Introduction

Intrinsically photosensitive retinal ganglion cells (ipRGCs), a unique population of mammalian retinal ganglion cells (RGCs), express the novel photopigment melanopsin and signal light directly [1,2,3,4]. These cells send their axons to hypothalamic suprachiasmatic nucleus (SCN), a site of the master biological pacemaker, and other non-image forming (NIF) visual centers, thus mediating a wide variety of physiological processes, such as photoentrainment of circadian rhythms, pupillary light reflex and nocturnal suppression of pineal melatonin secretion, etc. [5,6,7,8,9].

Activity of ipRGCs may also be subject to intra-retinal circadian modulation. In rat retina, the expression of melanopsin undergoes robust daily fluctuation, with peak levels of mRNA and protein of this molecule occurring at night [10,11,12,13,14]. Consistent with this, in rats kept in constant darkness, a modest but significant increase in ipRGC photoresponses has been observed in the subjective night, as compared with other circadian phases [15]. More recently, ipRGC-controlled human post-illumination pupil responses are shown to exhibit circadian changes in amplitudes [16]. However, relatively little is known about the mechanisms underlying the circadian modulation of ipRGC activity.

Activities of retinal neurons are modulated by melatonin (see [17,18] for reviews). Retinal melatonin is synthesized by photoreceptors in a circadian manner, being higher at night and lower during the daytime [19,20,21,22]. In mammalian retina, this neurohormone exerts its function via acting on two distinct subtypes of specific receptors, namely MT_1_ and MT_2_ receptors [23,24]. Specifically, in rat RGCs melatonin potentiates glycine receptor-mediated current of these cells via MT_2_ receptors [25].

Whilst melatonin receptors are known to be expressed in mammalian RGCs [26,27,28,29,30,31,32,33,34], very few data about the expression of these receptors on ipRGCs are now available. MT_1_-immunoreactivity has been detected in mouse ipRGCs [35], but whether MT_2_ receptor is also expressed by ipRGCs remains unclear. Moreover, most of the previous work concerning circadian modulation of ipRGCs has been conducted in rats [10,11,12,13,15]. Given the fact that the expression of melatonin receptors is highly species-dependent [17,18], we studied whether and how MT_1_ and MT_2_ melatonin receptors are expressed in a specific subtype (M1-type) of ipRGCs in rats using fluorescence double-staining technique. We demonstrated the co-existence of MT_1_ and MT_2_ receptors in all melanopsin-positive M1 ipRGCs, and the expression of MT_1_ and MT_2_ receptors in these cells could be seen as early as the day of birth (P0). These results suggest that melatonin may directly modulate the activity of rat ipRGCs by activating MT_1_/MT_2_ receptors.

## Materials and Methods

### Ethics statement

Use and handling of animals were strictly in accordance with the U.S. National Institutes of Health (NIH) guidelines for the Care and were approved by Institutional Animal Care and Use Committees of Fudan University. All efforts were made to minimize the number of animals used and their suffering.

### Animals

A total of 29 adult male Sprague-Dawley rats (SLAC, Shanghai, China) weighing 220–280 g, and 4 P0 newborn rats were used in this study. Adult animals were housed for at least 2 weeks in a 12-h light (~500 lux): 12-h dark (LD) cycle before experiments. To avoid possible diurnal impacts on protein expression, all retinas were harvested and fixed 8–10 hours after light onset (Zeitgeber Time 8–10).

### Antibodies

Melanopsin was probed with a polyclonal antibody raised in goats against a sequence between amino acids 410–460 of rat melanopsin (SC-26962, Santa Cruz Bio-technology, Santa Cruz, CA, USA; 1:50). It has been used previously to identify ipRGCs [36,37]. To label MT_1_ receptors, we used a rabbit polyclonal antibody directed against a peptide corresponding to a region of the third intracellular loop (residues 223–236: (C) RVKPDNKPKLKPQD) of mouse MT_1_ receptor (AMR-031, Alomone laboratories, Jerusalem, Israel; 1:500). The MT_2_ receptor antibody was raised in rabbits, targeting the third intracellular loop (residues 232–246: (C) RKAKATRKLRLRPSD) of the mouse MT_2_ (AMR-032, Alomone; 1:50). In some of our experiments, a rabbit anti-MT_2_ receptor antibody raised against N-terminal extracellular domain of human MT_2_ receptor (SAB2900212, Sigma, St. Louis, MO, USA; 1:200) was used to probe MT_2_ immunoreactivity. The secondary antibodies were Alexa Fluor 555-conjugated donkey anti-goat IgG (for melanopsin) and Alexa Fluor 488-conjugated donkey anti-rabbit IgG (for MT_1_/MT_2_) (Invitrogen, Carlsbad, CA, USA; 1:200). In the experiments demonstrating the cytoplasmic staining of melatonin receptors, Alexa Fluor 555-conjugated wheat germ agglutinin (WGA, Invitrogen; 10 μg/ml) was used to label the plasma membrane of retinal neurons.

### Immunohistochemistry

#### Immunofluorescent double-labeling of retinal sections

Animals were deeply anesthetized with 20% urethane (10 ml/kg) and enucleated, and then sacrificed by urethane overdose. Eye cups, made by removing the anterior part of the eyes, were immediately immersed in fresh 4% paraformaldehyde in 0.1 M phosphate buffered saline (PBS, pH 7.4) for 20 min. The eyecups were then chilled sequentially in 10% (w/v), 20% and 30% sucrose in 0.1 M PBS at 4℃, embedded in OCT (Miles Inc., Elkhart, IN, USA), and frozen by liquid nitrogen. A CM1950 cryostat (Leica Microsystems, Wetzlar, Germany) was used to cut 14 μm frozen sections, which were then mounted on gelatin chromium-coated slides and stored at -20℃. The sections were blocked in a medium containing 6% normal donkey serum and 0.2% Triton X-100 in 0.1 M PBS for 2 h at room temperature, and incubated with the primary antibodies in a buffer (3% normal donkey serum, 1% bovine serum albumin and 0.2% Triton X-100 in 0.1 M PBS) at 4℃ for 3 days. Binding sites of the primary antibodies were revealed by incubating with the fluorescent second antibodies for 2 h at room temperature. Staining by a mixture of two secondary antibodies after incubation with one of the two primary antibodies showed no cross-reactivity of species specific secondary antibodies. Control experiments were performed by pre-absorbing the antibodies for melatonin receptors with the corresponding immunizing peptide.

Immunofluorescence images were acquired using a Fluoview FV1000 confocal microscopes (Olympus Corporation, Tokyo, Japan) under a 60× oil-immersion objective lens (N.A.1.42). For each of double-labeling experiments, totally 30~80 sections on 4~10 different glass slides derived from four or five eyeballs were examined. To avoid any possible reconstruction stacking artifact, double-labeling was precisely evaluated by sequential scanning on single-layer optical sections. Images were resized and adjusted for global brightness and contrast in Photoshop CS3 (Adobe Systems, San Jose, CA, USA).

#### Immunofluorescent staining of ipRGCs in retinal whole-mounts

The retinas dissected from eye cups were flattened (ganglion cell side up), with the aid of four radial cuts, onto a piece of filter membrane (AABP02500, Millipore, Billerica, MA, USA), and fixed in 4% paraformaldehyde for 2 h. After a blocking step (5% normal donkey serum plus 1% Triton X-100 in 0.1 M PBS, 2 h, room temperature), the whole-mount retinas were incubated with the primary antibody against melanopsin for two days at 4℃. The retinas were then reacted with the secondary antibody overnight at 4℃, and finally mounted onto glass slides.

Immunofluorescence photomicrographs of flat-mounted retina were obtained on an Axioskop 40 microscope (Carl Zeiss Inc., Oberkochen, Germany) under a 20× objective lens. Dendritic profile reconstruction of individual ipRGCs followed the methods described by Berson et al. [38]. Briefly, in a single retinal region chosen randomly, a series of images at multiple focal depths were captured, so that melanopsin-positive area in this location was in sharp focus at least in one image. The images were then imported to Photoshop, with each image in one layer. With the ‘pencil’ tool, soma-dendritic profiles of one to several ipRGCs were traced continuously from one image to the next, on a separate overlying transparent layer. Morphometric measurements were performed with Image J (http://rsbweb.nih.gov/ij/). The contours of somata and the minimal convex polygon enclosing the dendritic field were traced to calculate equivalent diameters, so as to assess the size of the cell body and the dendritic area of each cell.

### Western blot analysis

Western blot analysis was performed referring to a previous study [39]. A membrane protein extraction kit (K268–50, BioVision, Milpitas, CA, USA) was used for extracting total cellular membrane proteins from rat retinas. Equivalent amounts of freshly extracted retinal lysate (10 μg/lane) were electrophoresed on 10% SDS-PAGE and then electrophoretically transferred onto PVDF membranes. Non-specific binding was blocked in a blocking solution (pH 7.6) containing 20 mM Tris-HCl, 137 mM NaCl, 0.1% Tween-20 (TBST) and 3% (w/v) bovine serum albumin for 2 h at room temperature. The blots were then incubated with the same antibody for melanopsin or melatonin receptors used in immunohistochemistry experiments (1:200 for melanopsin; 1: 4000 for MT_1_; 1: 2000 for MT_2_) overnight at 4℃, followed by horseradish peroxidase (HRP)-conjugated donkey anti-goat IgG (for melanopsin) or donkey anti-rabbit IgG (for MT_1_ and MT_2_) (both 1:4000, Santa Cruz Biotechnology) for 2 h at room temperature. Immunoblots were visualized by enhanced chemiluminescence (Amersham Biosciences, Piscataway, NJ, USA), and finally captured using ChemiDoc XRS System with Image Lab software (Bio-Rad, Hercules, CA, USA). To estimate the molecular weight (MW), a pre-stained protein ladder (26617, Thermo Scientific, Waltham, MA, USA) was used.

## Results

### Morphology of melanopsin-labeled rat M1 ipRGCs

In a recent work using a rabbit melanopsin antibody, three subclasses of rat ipRGCs were labeled [40]. We therefore first carried out a series of morphological analysis to determine which type (s) of rat ipRGCs could be stained by the goat antibody used. Even though this antibody has been reported to identify ipRGCs [36,37], we further examined the specificity of it using Western blot analysis. In rat retinal homogenates, the antibody against melanopsin recognized a single band at ~55 kDa (Fig. 1a1), which is comparable to that of vertebrate melanopsin [41,42]. No signal was detected when the antibody was omitted (Fig. 1a2). Fig. 1A is a photomicrograph taken with a fluorescence microscope, showing an array of immunofluorescent cell bodies at low density in the ganglion cell layer (GCL), in a flat-mounted retina labeled with this antibody. Meanwhile, a single plexus of melanopsin-positive dendritic processes, occupying the distal part of the inner plexiform layer (IPL), could be clearly seen (Fig. 1B), but there was no immunofluorescent dendritic network in the proximal part of the IPL. Occasionally, a very few number of somata, which may be those of displaced ipRGCs [4,38], appeared in the inner nuclear layer (INL) (Fig. 1C). Fig. 1D is the confocal micrograph of a retinal vertical section, showing a single layer of strongly labeled dendritic arbors located in the OFF sublamina in the IPL, which was immediately close to the IPL-INL border. The morphological features of the labeled cells, as shown in both the flat-mount and the section, were typical of the M1-type ipRGC, which is characterized by dendritic varicosities, as well as sparsely branched dendritic arbors monostratifying at the outmost of the IPL. It should be noted that all the cells labeled by this antibody showed similar morphological features, and they were morphologically distinct from M2~M5-type cells, which are either bistratified or mono-stratified in the ON sublamina of the IPL [43,44]. Based on a bulk of microphotographs taken along the Z-axis of the whole mount retina preparations, we reconstructed the dendritic profiles of 24 individual melanopsin-positive cells, chosen randomly from six retinas. Fig. 1E shows five cells with representative soma-dendritic structures. The major morphometric parameters of these cells, such as soma diameter (13.9±0.4 μm), dendritic field diameter (367.4±15.5 μm), primary dendrite number (2.7±0.1), terminal neurite tips number (11.5±1.0) and dendritic arbor branch point number (8.1±1.0), all correspond to those of previously reported “M1” cells or the subset of melanopsin-containing cells monostratified in the outermost of the IPL in rat [4,40,45,46] (Table 1).

**Table 1:** Comparison of the morphometric parameters of rat M1 cells in this work with those described in other studies*.

	Dendritic field diameter (μm)	Soma diameter (μm)	Primary dendrite	Branch points	Terminal neurite tips
This study^#^	367.4±15.5	13.9±0.4	2.7±0.1	8.1±1.0	11.5±1.0
Hattar et al., 2002	—	—	—	—	—
Li et al., 2006	—	—	~2.7	—	—
Ingham et al., 2009	—	15	—	—	—
Esquiva et al., 2013	~356.9	~12	—	~8.5	~12

*‘—’: data not available.

#Measurements are mean±SEM.

**Fig 1:**
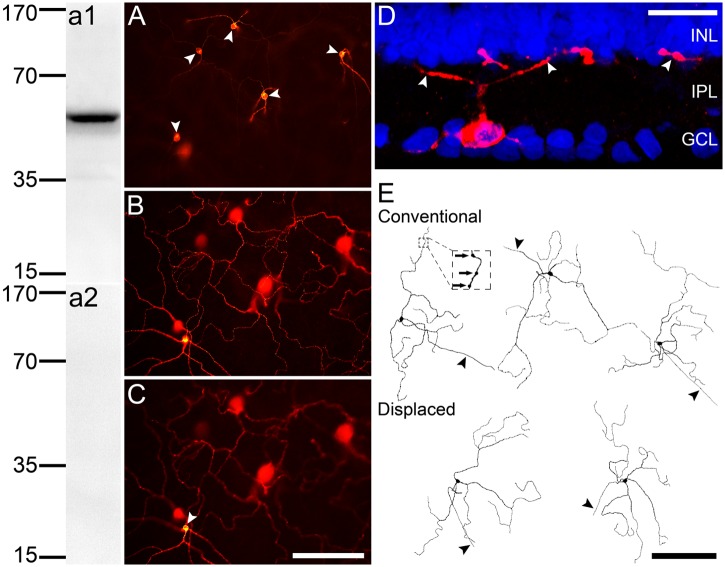
Melanopsin immunostaining reveals a single subclass of ipRGCs in rat retina. **(a1)** Western blot analysis of whole rat retina homogenates for the melanopsin antibody revealed a single band of ~55 kDa. **(a2)** The immunoblot signals were completely eliminated when the primary antibody was omitted. MW scale (kDa) is shown on the left. **(A-C)** Photomicrographs captured from the same area of a whole-mount retinal preparation, but at different focal planes. **(A)** Five melanopsin-positive somata (arrow heads) were identified in the GCL, corresponding to the ‘conventionally placed’ cells. **(B)** A strongly-labeled plexus of immunoractive dendritic processes, which is located at the outer limit of the IPL. The soma of one ‘displaced’ cell (arrow heads) is in sharp focus in **(C)**, which focuses on the INL. **(D)** A vertical section counterstained with DAPI (blue) to reveal cellular laminae of the inner retina, showing monotratified dendritic arborization (arrow heads) of the melanopsin-immunoreactive (red) cells at the boundary between the INL and IPL. **(E)** Representative examples demonstrating the morphological profiles of melanopsin-stained cells reconstructed from the whole-mount retinal preparations. Three conventionally located (***upper***) and two displaced cells (***lower***) are shown. The sparse dendritic branching pattern is similar among these cells, typical of M1-type ipRGCs. Arrows in the inset point to the apparent varicosity-like structures on the dendrites. Arrowheads indicate the axons. INL, inner nuclear layer; IPL, inner plexiform layer; GCL, ganglion cell layer. Scale bar is 200 μm in **(A-C, E)** and 20 μm in **(D)**.

### Co-expression of MT_1_ and MT_2_ receptors on rat M1 cells

**Fig 2:**
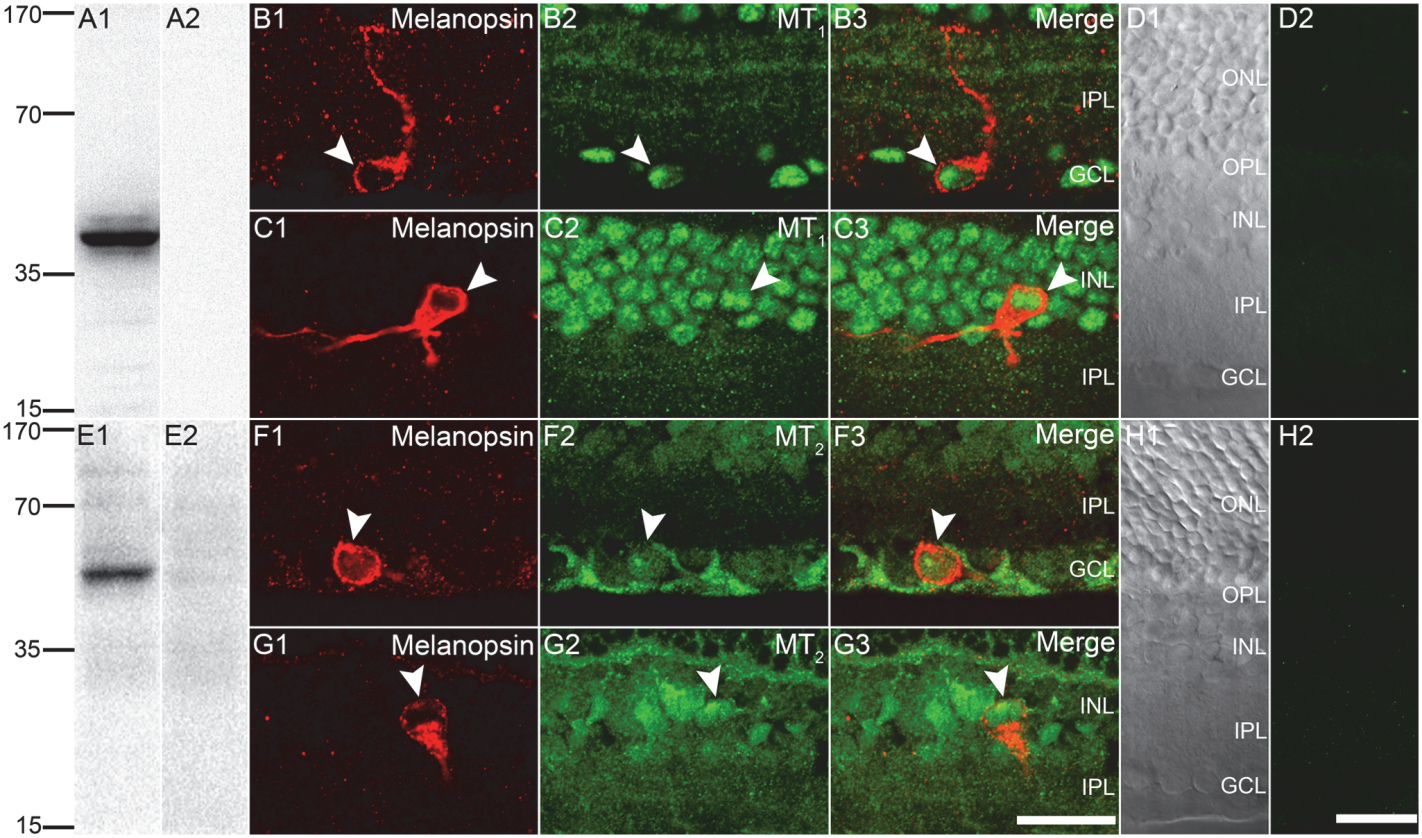
Expression of MT_1_ and MT_2_ receptors on rat M1 cells. **(A1)** Western blot analysis of retinal homogenates for the MT_1_ antibody revealed a single band at ~40 kDa. MW scale (kDa) is shown on the left. **(A2)** No band was detected when the MT_1_ antibody was pre-absorbed with the immunizing antigen. **(B1-B3)** and **(C1-C3)** Confocal fluorescence microphotographs of retinal sections, double labeled by melanopsin and MT_1_. The cytoplasm of a conventionally placed M1 cell **(B1)**, and a displaced M1 cell **(C1)** are both MT_1_ immunoreactive. Note that, several faint MT_1_-immunoreactive strata could be seen in the IPL **(B2** and **C2)**. **(D1**) Nomarski image showing multiple layers of a retinal section. **(D2)** Same retinal section as in **(D1)**, showing that no immunofluorescence labeling was present when the MT_1_ antibody was pre-absorbed with the immunizing antigen. **(E1)** Western blotting of retinal homogenates for the MT_2_ antibody revealed a single band at ~45 kDa, as expected for MT_2_. MW scale (kDa) is shown on the left. **(E2)** No band was seen when the MT_2_ antibody was pre-absorbed with the blocking peptide. **(F1-F3)** and **(G1-G3)** Confocal fluorescence microphotographs of retinal sections, showing colocalization of melanopsin and MT_2_ immunoreactivity. The cytoplasm of a conventionally placed M1 cell **(F1)** and a displaced M1 cell **(G1)** are both stained by MT_2_. **(H1)** Nomarski image showing retinal layers more clearly. **(H2)** Same retinal section as in **(H1)**, which was treated with the immunofluorescence labeling procedure for MT_2_, but the MT_2_ antibody was pre-absorbed with the immunizing antigen. Double labeled cells are indicated by arrow heads. Scale bars = 20 μm.

The specificity of the MT_1_ receptor antibody was assessed using Western blot analysis. In rat retinal homogenates, this antibody revealed a single predominant band at approximately 40 kDa (Fig. 2A1), corresponding to the known molecular weight of the mammalian MT_1_ receptor [23,35]. No such band was detected when the antibody was pre-absorbed with the immunogen peptide (Fig. 2A2). These data indicated that the protein recognized by the antibody might indeed be the MT_1_ receptor. Fig. 2B1-B2 show vertical cryostat sections of the rat retina, doubled labeled by antibodies against melanopsin and MT_1_. MT_1_ receptor immunoreactivity was clearly seen in many cells in the GCL, in addition to the strong labeling in the INL and diffuse staining in the IPL. Notably, most cells in the ONL and a few cells in the INL were MT_1_-negative (data not shown). Some of the MT_1_-positive cells in the GCL were also immunoreactive with melanopsin (Fig. 2B3). As clear from the merged image (Fig. 2B3), the labeling for MT_1_ appeared to be restricted to the cytoplasm. A few of melanopsin-positive M1 cells were displaced to the INL (Fig. 2C1), and these cells were also MT_1_-immunoreactive (Fig. 2C2, C3). When the antibody for MT_1_ was pre-absorbed with the blocking peptides (negative control), only low level of background was detected (Fig. 2D2), which was similar to the no primary antibody control (data not shown), confirming that the labeling was specific. The Nomarski image of Fig. 2D2 (Fig. 2D1) shows retinal layers more clearly.

In another independent set of experiments, we also detected immunopositive signals for MT_2_ in melanopsin-positive M1 cells. The AMR-032 rabbit anti-MT_2_ antibody generated a single band of appropriate size (~45 kDa) [24,25] by Western blotting of rat retina homogenates (Fig. 2E1). Pre-incubation of the antibody with its target peptide abolished all the blots (Fig. 2E2), suggesting the specificity of the antibody. Just like observed for MT_1_ receptor, immunoreactive signals for MT_2_ receptor were clearly seen in the cytoplasm of melanopsin-positive M1 cells, residing either in the GCL (Fig. 2F1-F3) or in the INL (Fig. 2G1-G3). The immunofluorescence staining for MT_2_ was abolished when the primary antibody was treated with immunizing antigen (Fig. 2H1 and H2). Another rabbit anti-MT_2_ antibody used in the present work, SAB2900212, generated a staining pattern highly comparable to that of AMR-032, and some cells stained by this antibody were also co-labeled by melanopsin (data not shown).

**Fig 3:**
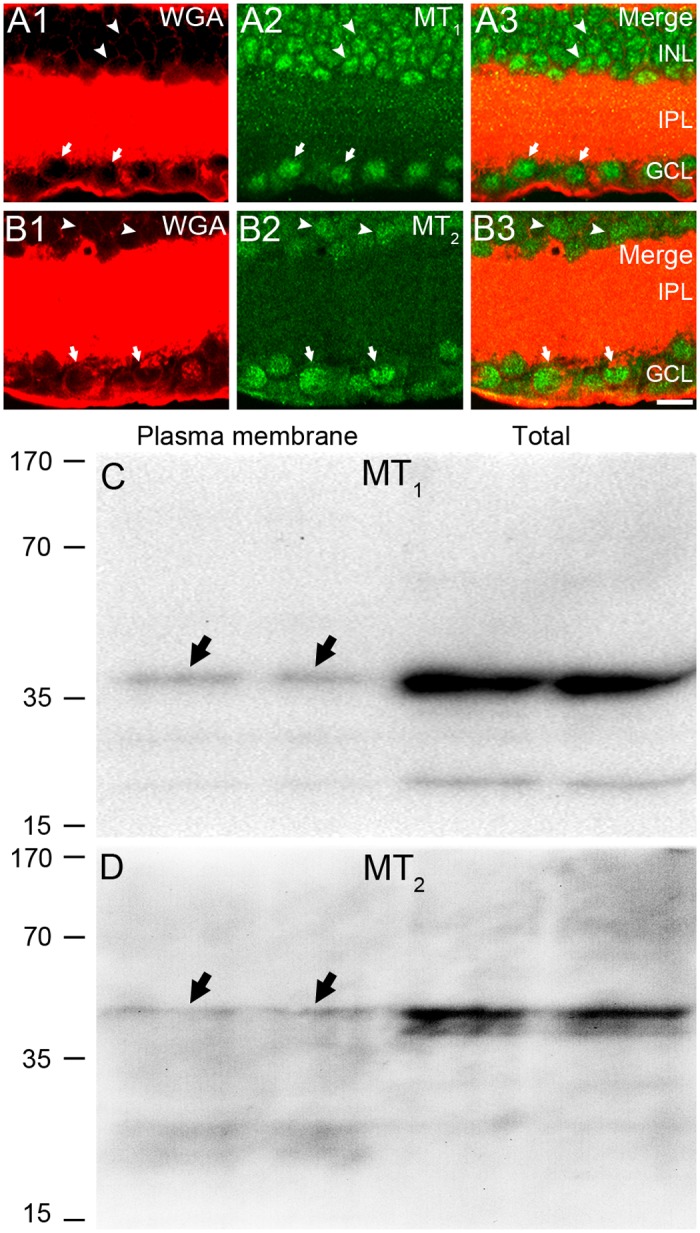
Evidence of cytoplasmic localization of melatonin receptors. **(A)** and **(B)** Double immunolabeling of rat retina cryostat sections using MT_1_/MT_2_ antibody and Alexa Fluor 555-conjugated WGA. In a vast majority of neurons in the INL and GCL, granular signals for either MT_1_ or MT_2_ are both closely circumscribed by the WGA-labeled cell membrane, suggesting the cytoplasmic staining. Scale bar = 10 μm. **(C)** and **(D)** A comparison of the results yielded by Western blot analysis for the MT_1_/MT_2_ antibodies using retinal plasma membrane extracts (left two columns) and whole retinal homogenates (right two columns). The MT_1_ and MT_2_ antibodies both generated a band of appropriate size (40–45 kDa). Note that bands for membrane extracts (arrows) were much lighter than those for whole retinal homogenates.

The cytoplasmic staining of both melatonin receptors was further confirmed by a double-staining control experiment using the MT_1_/MT_2_ antibody and fluorophore-conjugated WGA (a well-established cell membrane marker [47]). As shown in Fig. 3A1-A3 and B1-B3, in a vast majority of retinal neurons examined, the area circumscribed by WGA-positive signals was fully filled by the MT_1_/MT_2_ labeling, thus providing direct evidence for the cytoplasmic labeling for MT_1_/MT_2_ receptors. One may argue that staining for MT_1_/MT_2_ should be limited to the membrane given the fact that melatonin receptors are thought to be membrane receptors. It is likely that the cytoplasmic staining in the present work may be largely because the antibodies used preferentially recognize non-membrane-located melatonin receptors. This possibility was strengthened by making a comparison between the results yielded by Western blot analysis for the MT_1_/MT_2_ antibodies using retinal plasma membrane extracts, and those using whole retinal homogenates (Fig. 3C and D). The bands obtained with retinal membrane extracts were found to be much weaker, as compared with those obtained with retinal homogenates.

To determine whether MT_1_ and MT_2_ receptors may be co-expressed in a single M1 ipRGC, we performed a quantitative analysis of the expression of these two receptors in two populations of melanopsin-positive M1 neurons. For the first population, 110 M1 cells, including 102 conventional and 8 displaced cells, were collected from 104 retinal cross sections double labeled by antibodies against melanopsin and MT_1_. Labeling for both melanopsin and MT_1_ was seen in all these cells without exception. For the second population, in a total of 20 M1 cells, including 19 conventional cells and one displaced cell, collected from 16 retinal sections, it was also found that none of these cells were not MT_2_-immunoreactive. Based on these quantitative data, we derived that both MT_1_ and MT_2_ are co-expressed in virtually all M1 cells.

### Expression of melatonin receptors on neonatal ipRGCs

**Fig 4:**
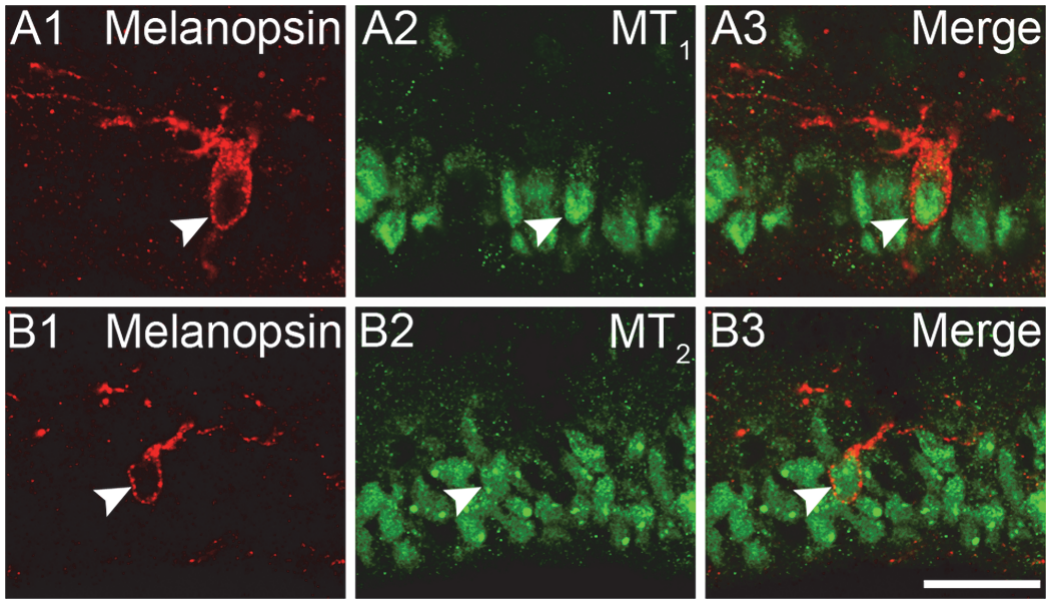
Neonatal expression of melatonin receptors on rat ipRGCs. **(A1-A3)** Confocal fluorescence microphotographs of a rat retinal section harvested at P0, showing that MT_1_ receptor is immunohistochemically localized to the cytoplasm of a melanopsin-positive cell (arrow head). **(B1-B3)** Confocal fluorescence microphotographs of P0 rat retina, double labeled by melanopsin and MT_2_. MT_2_ immunoreactivity was detected in the cytoplasm of a melanopsin-expressing cell (arrow head). Scale bar = 20 μm.

ipRGCs are likely the first functional photosensitive neurons in the retina, sensing light immediately after the birth [48,49]. There is convergent evidence that ipRGCs mediate a number of light-driven developmental functions during the early postnatal stage (see Discussion). It is plausible that these functions may be subject to melatonin-mediated modulation even in neonatal animals. To explore this possibility, we performed double-labeling analysis on retinal sections harvested from P0 rats. In these rats the melanopsin antibody stained a small set of cells with their somata located in the GCL (Fig. 4A1 and B1). Double-labeling analysis revealed that these melanopsin-positive neonatal ipRGCs were invariably double-labeled by MT_1_ (Fig. 4A2, A3) or MT_2_ (Fig. 4B2, B3), even though most of them were not yet showing dendritic stratifying feature typical of M1 cells. Quantitative analysis was also made for the expression of these two receptors on neonatal M1 cells (30 sections, 127 cells for MT_1_; 30 sections, 69 cells for MT_2_). It is likely that MT_1_ and MT_2_ receptors are co-expressed in ipRGCs in neonatal animals, as early as the day of birth.

## Discussion

### Co-expression of MT_1_ and MT_2_ receptors on ipRGCs

MT_1_ receptor protein and mRNA are localized to RGCs in a variety of species, including human, monkeys, guinea pigs, mice and rats [26,27,28,29,31,32,33,34,35]. Immunoreactivity for MT_2_ is also seen in RGCs of human and rat [25,30] (but MT_2_ transcripts are recently reported to be absent in the GCL of mouse [34]). However, information regarding the expression of melatonin receptors on ipRGCs is rather scant, largely because most of earlier studies on retinal localization of melatonin receptors had been done before ipRGCs were discovered. Recently, using immunohistochemical staining, Sengupta and colleagues reported that MT_1_ receptors were expressed in mouse ipRGCs [35], but these authors did not describe the MT_1_ expression pattern in detail nor did they evaluate MT_2_ receptors. Furthermore, whether these two subtypes of melatonin receptors may be co-expressed in these cells remained to be addressed. In the present work, we demonstrated the co-expression of MT_1_ and MT_2_ receptors in M1-type ipRGCs specifically labeled by the antibody against melanopsin. The labeling for MT_1_ and MT_2_ was restricted to the ipRGC cytoplasm, which is in agreement with several previous studies conducted in multiple types of central neurons [50,51,52,53]. There is also evidence that both MT_1_ and MT_2_ receptors may internalize from the cell membrane and accumulate in the cytoplasm under physiological conditions [47,54].

In regard to the specificity of the antibodies, in addition to the Western blot analysis, demonstrating that they recognized proteins of appropriate molecular weight (Fig. 2A and E), we also found that in sections of SCN, a structure known to express melatonin receptors, they produced staining patterns similar to those reported previously [50,55]. It is of interest that the labeling for melatonin receptors in SCN cells also appeared to localize to the cytoplasm, rather than to the membrane (S1 Fig.).

### Physiological implication

Melatonin modulates the activity of neurons in both outer and inner retina by distinct intracellular mechanisms due to activation of MT_1_ and MT_2_ receptors (for reviews, see [17,56]). It has been shown that melatonin may modulate the activity of neurons by changing the intrinsic excitability of these cells, including resting potentials [57,58], membrane conductances [59], and multiple types of membrane channels [60,61] etc., and/or synaptic transmission [61,62]. In rats, melatonin potentiates inputs from glycinergic amacrine cells to RGCs [25]. For ipRGCs, melatonin may modulate, just like at any conventional RGCs, synaptic input from bipolar and amacrine cells [63,64]. Alternatively, activation of melatonin receptors in these cells may directly intervene with the melanopsin phototransduction cascade. For example, melanopsin levels may be regulated by melatonin. It is also possible that some effectors of melatonin receptor activation, such as PLC and PKC, may be key components of the melanopsin cascade [65,66]. In addition, multiple phosphorylation sites melanopsin possesses [42,67] may also be the substrates of some kinases at the downstream of melatonin receptor activation. Melatonin may directly be involved in modulation of ipRGC activity by activating MT_1_ and MT_2_ receptors in these cells, thus regulating NIF visual function. It will be also of interest to explore how MT_1_ and MT_2_ receptors in ipRGCs could work in concert to mediate such modulation.

### Neonatal expression of melatonin receptors on ipRGCs

This work also demonstrated that MT_**1**_ and MT_2_ receptors are expressed in ipRGCs as early as P0. This result is not consistent with the observation by Fujieda et al. [27], which showed the absence of immunoreactivity for MT_1_ in early postnatal rat retinas, probably due to the less sensitive detection methodology used in that study. Our result conforms to multiple lines of evidence, which suggest a functional, although perhaps not completely mature, melatonin system available in neonatal animals. First, melatonin-synthesizing enzymes, N-acetyltransferase (NAT) and hydroxyindole-O-methyltransferase (HIOMT), are found active in fetal and postnatal brain [68]; NAT mRNA is also detectable in rat retina at P2 [69]. Secondly, melatonin content can be detected in both embryonic and neonatal brain [68]. Thirdly, mRNA levels of melatonin receptors in both the retina and brain in rats become measurable as early as E14 [68,70]. Finally, melatonin synthesis is already rhythmic at early developmental stage [69,71,72], and is capable of exerting various physiological functions [73,74].

Melanopsin gene expression precedes that of rod/cone opsins [75], and ipRGCs are actually the first functional retinal photoreceptors [48,49,76]. The early expression of melatonin receptors may modulate neonatal ipRGC activity in a diurnal and/or circadian manner, thus aiding in refining the melanopsin-mediated developmental events, such as modulation of retinal waves [77,78], light aversive behavior [79,80], determination of retinal neuron number [81], developmental patterning of ocular blood vessels [81] and formation of an inner-retinal photosensitive network before eye opening [49].

## Supporting Information

S1 Fig Melatonin receptor immunostaining in rat SCN sections. Both the MT_1_ **(A)** and MT_2_ **(B)** antibodies labeled the SCN neurons immunohistochemically, and the staining patterns were comparable with those reported previously. Note that the staining was localized to the cytoplasm but not to the cell membrane. Scale bar = 10 μm.
(TIF)
