# Supplementary figures and images for: Co-Expression of Two Subtypes of Melatonin Receptor on Rat M1-Type Intrinsically Photosensitive Retinal Ganglion Cells
Source: PLoS One. 2015 Feb 25;10(2):e0117967. doi: 10.1371/journal.pone.0117967 (PMC4340921; doi:10.1371/journal.pone.0117967)

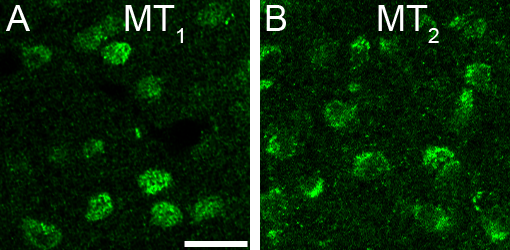

Supplement: S1 Fig — Both the MT1 (A) and MT2 (B) antibodies labeled the SCN neurons immunohistochemically, and the staining patterns were comparable with those reported previously. Note that the staining was localized to the cytoplasm but not to the cell membrane. Scale bar = 10 μm. (TIF) [file pone.0117967.s001.tif]
